# Targeting newly identified ERβ/TGF‐β1/SMAD3 signals with the FDA‐approved anti‐estrogen Faslodex or an ERβ selective antagonist in renal cell carcinoma

**DOI:** 10.1002/1878-0261.12377

**Published:** 2018-10-30

**Authors:** Wenbin Song, Dalin He, Yule Chen, Chiuan‐Ren Yeh, Iawen Hsu, Qingbo Huang, Xu Zhang, Luke Sien‐Shih Chang, Li Zuo, Jiasheng Chen, Karen M. Doersch, Chawnshang Chang, Lei Li, Shuyuan Yeh

**Affiliations:** ^1^ Department of Urology The First Affiliated Hospital Xi'an Jiaotong University China; ^2^ George Whipple Lab for Cancer Research Departments of Urology and Pathology University of Rochester Medical Center NY USA; ^3^ Department of Urology Chinese PLA General Hospital Beijing China

**Keywords:** epithelial‐mesenchymal transition, estrogen receptor β, ICI 182,780, selective estrogen receptor modulator, tamoxifen, TGFβ

## Abstract

Renal cell carcinoma (RCC) has the third highest mortality rate among urological tumors, and 20–30% of RCC patients present with metastatic RCC at the time of diagnosis. Although recent studies have indicated that estrogen receptor β (ERβ) could play promoting roles in RCC progression, the detailed mechanisms remain to be clarified. In the present study, we found that expression of ERβ, but not ERα, increases with tumor stage and grade, and also observed that modification of ERβ signals using estrogens/anti‐estrogens, shRNA knockdown of ERβ and overexpression of ERβ using ectopic cDNA affects RCC cell proliferation, migration and invasion. Mechanism analysis revealed that ERβ can promote RCC cell invasion via an increase in transforming growth factor β1 (TGF‐β1)/SMAD3 signals, and interrupting TGF‐β1/SMAD3 signals with a TGFβR1 inhibitor can reverse/block ERβ‐increased RCC cell migration. Importantly, preclinical analyses using *in vivo* mouse models of RCC revealed that targeting of this newly identified ERβ/TGF‐β1/SMAD3 pathway with either the FDA‐approved anti‐estrogen ICI182,780 (Faslodex) or a selective ERβ antagonist 4‐[2‐phenyl‐5,7 bis(trifluoromethyl)pyrazolo[1,5‐a]pyrimidin‐3‐yl]phenol can significantly reduce RCC tumor growth and invasion, which may be suitable as the basis for novel therapies to more effectively suppress metastatic RCC.

**Abbreviations**
ccRCC: clear cell renal cell carcinoma
E2: 17 β‐estradiol
EGF: epidermal growth factor
EMT: epithelial–mesenchymal transition
ER: estrogen receptor
ESR2: estrogen receptor β
ICI: ICI182, 780
IF: immunofluorescence
IGF‐1: insulin‐like growth factor‐1
IHC: immunohistochemical staining
MTT: 3‐(4,5‐dimethylthiazol‐2‐yl)‐2,5‐diphenyl‐tetrazolium bromide
PHTPP: 4‐[2‐phenyl‐5,7 *bis*(trifluoromethyl)pyrazolo[1,5‐*a*]pyrimidin‐3‐yl]phenol
RCC: renal cell carcinoma
TCGA: The Cancer Genome Atlas
TGF‐β1: transforming growth factor β1
TGFβR‐1: transforming growth factor β receptor‐1

## 1 Introduction

Renal cell carcinoma (RCC) accounts for approximately 3% of adult malignancies and is the third leading cause of death among urological tumors (Comperat and Camparo, 2012). Worldwide incidence and mortality rates of RCC are steadily rising at a rate of approximately 2–3% per decade (Gupta *et al*., 2008). It is estimated that approximately 25–30% of RCC patients have metastases at the time of diagnosis. Even after resection of the primary tumor by radical or partial nephrectomy, relapse occurs in 20–30% of RCC patients (Whelan, 2003). To date, the etiological factors of RCC remain largely unknown (Murai and Oya, 2004). In addition to obesity, diabetes and hypertension, other known risk factors include cigarette smoking and diet, as well as oophorectomy and parity in women (Asal *et al*., 1988).

Estrogens regulate the growth, development and functioning of both the normal and cancerous breast (Russo and Russo, 1997). There are two subtypes of estrogen receptor (ERs), ERα and ERβ. ERα and ERβ diverge early during evolution (Kelley and Thackray, 1999) and differ mostly in the N‐terminal A/B domain and, to a lesser extent, in the ligand binding domain (E domain). Although the structures of ERα and ERβ are similar, their histological distributions and biological functions are not the same. Distribution of ERα and ERβ varies in different tissue types (Fox *et al*., 2008). Our new data showed that ERβ is positively, although ERα is negatively, expressed in RCC cell lines and tissues. Thus, we focused on studying the roles of ERβ in RCC development.

ERβ plays multiple physiological roles in the human body and previous studies indicate that ERβ differentially expresses in various normal and cancerous tissues (Han *et al*., 2002; Rozati *et al*., 2002; Schneider *et al*., 1979; Zhou *et al*., 2001). To date, several studies show that the ERβ expression is decreased in breast, as well as colon and ovarian cancer (Campbell‐Thompson *et al*., 2001; Iwao *et al*., 2000), suggesting that ERβ could function as an inhibitor of tumor initiation in these cancers.

In urinary tract tumors, intra‐prostatic metabolism of testosterone to estrogens has been recently proposed to play a vital role in the regulation of prostate gland growth (Muthusamy *et al*., 2011). ERβ is detected in the prostate gland (Kuiper *et al*., 1996), and estrogen signaling driven by ERβ has been shown to negatively regulate prostate gland growth (Carruba, 2007). Interestingly, in bladder cancer, reports have suggested that ERβ could play positive roles in promoting bladder cancer progression and sh‐ERβ or an ERβ antagonist could inhibit bladder cancer growth (Hsu *et al*., 2014). Our recent study showed that infiltrated immune cells could alter ERβ to promote RCC progression (Song *et al*., 2015). Furthermore, *in vivo* animal results indicated that supplementation of the synthetic estrogen, diethylstilbestrol, could induce RCC development (Wolf *et al*., 1998). Our recent reports suggested that ERβ could modulate the functions of circular RNA‐ATP2B1, and long non‐coding RNA‐HOTAIR to promote RCC progression (Ding *et al*., 2018; Han *et al*., 2018). However, how ER directly regulates RCC progression and the underlying mechanisms remain an under‐investigated area that requires further investigation.

In the present study, we provide evidence showing that there is little ERα in kidney or RCC tissues, as well as increased ERβ expression in the tumors at later stages of RCC. The increased ERβ is associated with a worse survival for RCC patients. Further analyses using *in vitro* cell studies and mouse RCC models showed that estrogens function via ERβ to promote the proliferation, migration and invasion of RCC. In addition, our data confirm that ERβ affected the expression of transforming growth factor β1 (TGFβ1)/SMAD3 signals to control RCC invasion. Targeting ERβ/TGFβ1/SMAD3 signals with FDA‐approved anti‐estrogens could help in the development of new therapies to better treat RCC.

## 2 Materials and methods

### 2.1 RCC tissue samples for immunohistochemical staining (IHC) and RNA analysis

**Figure 1:**
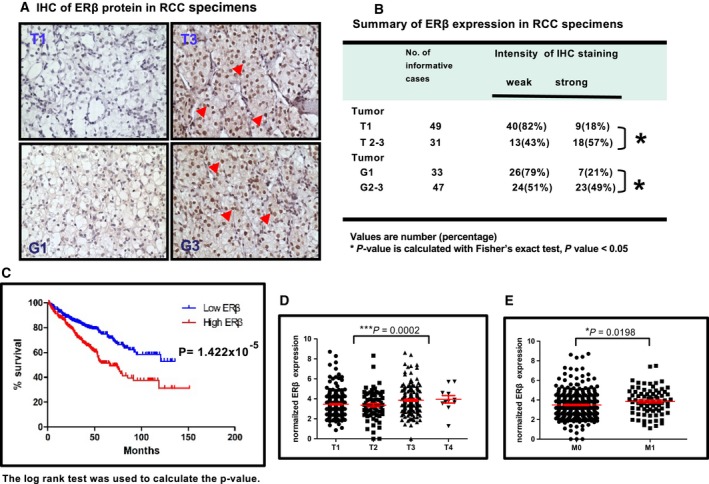
Higher expression of ERβ was associated with a poor prognosis in ccRCC patients. (A) IHC staining of ERβ expression in low and high stages or grades of 80 human RCC specimens. The ERβ showed nuclear staining signals (arrows). Higher ERβ signals were detected in T3/G3 RCC patient samples. (B) IHC of ERβ protein levels in different stages or grades of RCC tissues. T2‐3 RCC tissues (57%) showed a significantly higher ERβ‐positive rate compared to T1 tissues (18%). Similarly, G2‐3 RCC tissues (49%) showed a significantly higher ERβ‐positive rate compared to G1 tissues (21%) (*P = 0.0184). (C) Overall survival curve of ccRCC patients according to 50% patients with higher vs. 50% patients with lower ERβ mRNA expression based on TCGA database. (D, E) The correlation between ERβ mRNA expression and pathological stage of tumor of ccRCC patients with completed follow‐up records (*n* = 518). All *P* vales are shown in the figure.

We obtained 80 paraffin‐embedded ccRCC specimens from 52 male and 28 female patients; 30 adjacent normal kidney tissues; and six metastatic specimens from four male and two female patients between January 2002 and March 2012 from the files of the Department of Urology, the First Affiliated Hospital of Medical College of Xi'an Jiaotong University for analysis. For the RNA sample collections used in Fig. 1SA, 119 cases of RNA samples from different grade RCC samples tissues were obtained postoperatively from the Department of Urology, Chinese People's Liberation Army General Hospital. The tumor areas were identified by two separate senior pathologists and were staged based on the 2011 Union for International Cancer Control (UICC) TNM Classification of malignant tumors.

The ethics of using human tissues were approved by the Review Board of the First Affiliated Hospital of Medical College of Xi'an Jiaotong University and the Review Board of the Chinese People's Liberation Army General Hospital. All patients provided their written informed consent for use of their tissue specimens. The study methodologies conformed to be standards set by the *Declaration of Helsinki*.

### 2.2 Analysis of the correlation of ERβ expression level and RCC overall survival rate

We analyzed the survival and RNAseq data of 537 ccRCC patients from The Cancer Genome Atlas (TCGA) database (http://cancergenome.nih.gov) using the LinkedOmics (http://linkedomics.zhang-lab.org/admin.php/). The TGCA Kidney renal clear cell carcinoma (KIRC) database contained information from 537 patients mainly with clear cell RCC. Among those 637 patients, there were 518 patients who had completed follow‐up records eligible for the overall survival analysis. Then, we used ESR2 (ERβ) plus TCGA clinical database as filters to perform correlative analysis of ERβ mRNA levels and survival outcomes in ccRCC patients. Based on ERβ mRNA levels, we separated those ccRCC patients into two groups: 50% with higher and 50% with lower ERβ expression. The results indicated that the 5‐year survival was worse in 50% of patients with higher ERβ mRNA expression.

### 2.3 Cell culture

RCC cell lines, 786‐O and A498, were purchased from the ATCC (Manassas, VA, USA) in 2013. After cells were received, each cell line was passaged and fifty ampules of cell stock frozen in liquid N_2_ within the first three passages. After an ampule was thawed, cells were used for the designed experiments within 15 passages, and maintained in Dulbecco's minimum essential medium (Invitrogen, Carlsbad, CA, USA) with 10% fetal bovine serum.

### 2.4 Lentiviral vector construction and virus production

Lentiviral shERβ (PLKO.1‐puro‐shERβ) plasmids were constructed with target sequences 5′‐GCGAGTAACAAGGGCATGGAA‐3′ or 5′‐CTTCAAGGTTTCGAGAGTTAA‐3′. Lentiviral particles were generated by calcium phosphate transfection of lentiviral expressing plasmids, packaging plasmid psPAX2 and envelope plasmid pMD2.G into HEK 293 cells, and the lentiviral particles were collected to transduce target cells in accordance with the manufacturer's instructions (Addgene, Cambridge, MA, USA). ERβ knockdown in 786‐O and ERβ overexpression in A498 cells were achieved by transducing cells with lentiviral ERβ small interfering RNA or ERβ cDNA, respectively.

### 2.5 Cell proliferation assay

RCC cells were seeded in 24‐well plates (5000 cells per well) and cultured for 0, 2, 4 and 6 days. Cells were harvested, lysed and stained with 3‐(4,5‐dimethylthiazol‐2‐yl)‐2,5‐diphenyl‐tetrazolium bromide (MTT) (Sigma‐Aldrich, St Louis, MO, USA) at 37 °C for 3 h. *OD*_570_  was measured to determine cell proliferation rate.

### 2.6 Colony formation assay

Cells were digested using trypsin/EDTA and 1 × 10^3^ cells were seeded into 6 cm plates and incubated at 37 °C at 5% CO_2_ for 10 days. Supernatants were discarded and then cells were rinsed in PBS twice and fixed in methanol for 10 min. The cells were stained with Giemsa's solution (AppliChem, St Louis, MO, USA) and air‐dried at room temperature. The experiments were triplicated and numbers of colonies (≥ 50 cells) were counted.

### 2.7 Cell migration and invasion assays

The migration or invasion capability of RCC cells was determined using a transwell assay. In brief, 1 × 10^5^ (invasion assay) or 2–5 × 10^4^ (migration assay) cells were seeded with serum‐free media into the upper chambers coated with matrigel (BD Corning, Corning, NY, USA) for invasion assays, or without coating for the migration assays. The migrated or invaded cells were fixed with 4% paraformaldehyde and stained with 1% toluidine blue. Cell numbers were counted in five randomly chosen microscopic fields (100×) per membrane. In some experiments, cells were treated with 10 μm of TGFβR‐1 inhibitor (SB525334; Selleckchem, Houston, TX, USA) for 48 h. All experiments were performed in triplicate each time, and experiments were repeated at least three times independently. The data were quantified from five representative fields and averaged. Data represent the mean ± SD of three independent experiments.

### 2.8 RNA extraction and quantitative real‐time PCR analysis

Total RNA was isolated using Trizol reagent (Invitrogen) in accordance with the manufacturer's instructions. Total RNA (2 μg) was subjected to reverse transcription using iScript™ Reverse Transcription Supermix (Bio‐Rad, Hercules, CA, USA). RT‐PCR has been described previously (Miyamoto *et al*., 2007; Slavin *et al*., 2014). The reverse transcription of miRNA was performed using the TaqMan miRNA Reverse Transcription Kit. Quantitative real‐time PCR was conducted using a SYBR CFX 96 system (Bio‐Rad).

### 2.9 Western blot analysis

The expression of ERs was determined by western blot analysis as described previously (Hsu *et al*., 2014; Song *et al*., 2015). Rabbit anti‐ERβ polyclonal antibody (GTX 110607; GeneTex, Irvine, CA, USA), mouse anti‐GAPDH monoclonal antibody (Santa Cruz Biotechnology, Santa Cruz, CA, USA), rabbit anti‐TGFB1 polyclonal antibody (Abcam, Cambridge, MA, USA) and mouse anti‐SMAD3 monoclonal antibody (Abcam) were used all at a dilution of 1 : 1000 in blocking buffer. The western blot analysis was scored positive if the band of interest was present at the expected molecular weight appropriate for each marker protein. All analyses were performed at least in triplicate.

### 2.10 Subcutaneous and renal capsule implantation of RCC cells

The transformed human RCC cells, 786‐O sh‐ERβ/sh‐Luc or A498 ERβ/Vec, were implanted under the renal capsule and subcutaneously in 8‐week‐old nude male or female mice. All tumor cells were stabilized with Luciferase for monitoring tumor growth and metastasis using *in vivo* image system (IVIS). At the end of experiments, the primary and metastatic tumors were harvested, measured, photographed and fixed for further histopathological analysis.

### 2.11 PHTPP, ICI182,780 and tamoxifen therapy effects on *in vivo* mouse RCC models

Luciferase‐labeled 786‐O cells were implanted under the renal capsule of 8‐week‐old female nude mice. Two weeks after implantation, the mice were randomly divided into different groups for treatment with dimethylsulfoxide, 4‐[2‐phenyl‐5,7 *bis* (trifluoromethyl) pyrazolo [1,5‐*a*]pyrimidin‐3‐yl]phenol (PHTPP), and ICI182,780 (ICI) were obtained from Tocris (Ellisville, MO, USA). Tamoxifen was purchased from Sigma Aldrich Ltd (St Louis, MO, USA). 10 μL of 1 × 10^−2^ m PHTPP, ICI or tamoxifen was mixed with 90 μL of sesame oil and injected intraperitoneally into each mouse, every other day for 4 weeks in total. All animal studies were approved by the Medical Experimental Animal Care Commission of at University Rochester Cancer Center, Rochester, NY, USA.

### 2.12 IHC and immunofluorescence staining (IF)

IHC and IF experiments were carried out as described previously (He *et al*., 2014; Miyamoto *et al*., 2012). Tumor sections were placed on slides and were incubated with the following primary antibodies, mouse monoclonal to ERβ (14C8, Abcam; dilution 1 : 50), rabbit anti‐TGFβ1 polyclonal antibody (ab27969, Abcam; dilution 1 : 100), N‐cadherin rabbit mAB (D4R1H, Cell Signaling Technology, Beverly, MA, USA; dilution 1 : 200) and anti‐luciferase (Santa Cruz Biotechnology) in 3% BSA in PBS overnight at 4 °C, followed by appropriate secondary antibodies. The stained slides were mounted and visualized by a bright‐field microscope or a fluorescence microscope.

### 2.13 Evaluation of Immunohistochemistry

Staining was examined at a high‐power field (400×) by two independent pathologists. The results obtained from five random fields were averaged. Each field was judged by a scoring system combining intensity and percentage. In each field, staining intensity was scored as: 0, no staining; 1, weakly positive staining; 2, moderately positive staining; 3, strongly positive staining (He *et al*., 2014; Miyamoto *et al*., 2012). The staining percentage of the relative number of cells stained was graded as: 0 for 0%, 1 for ≤ 25%, 2 for 25–50%, 3 for 50–75% and 4 for ≥ 75%. The total score of each section was calculated by multiplying the intensity and percentage scores. Sections with total scores ≥ 4 were defined as strong positive expression and < 4 as weak expression.

### 2.14 Statistical analysis

All quantitative data are presented as mean ± SD. Statistical significance among control group and treated groups was tested by ANOVA. *P* < 0.05 was considered statistically significant.

## 3 Results

### 3.1 ERβ and not ERα expression increased with RCC tumor stage and grade

To investigate the roles of ERs in RCC progression, we first applied the IHC with ERα and ERβ antibodies to stain ERs on 80 formalin‐fixed human RCC specimens from patients with either high or low stage/grade RCC tumors. The results revealed positive staining for the ERβ, and not ERα, in these RCC tumors. Importantly, we found that 18% (nine of 49) pT1 stage RCC tumors vs. 57% (18 of 31) of pT2‐3 stage RCC tumors had a stronger ERβ staining (*P* = 0.0005) (Fig. 1A). A similar conclusion was also made when we compared ERβ staining in different pathological grades; the results showed that 21% (seven of 33) Fuhrman's grade 1 (G1) RCC tumors vs. 49% (23 of 47) grade G2‐3 RCC tumors have higher and stronger ERβ IHC staining signals (*P* = 0.0184) (Fig. 1B). The results from a mRNA quantitative PCR assay of 119 RCC specimens also showed that ERβ mRNA expression increased with grade increases (Fig. S1A).

Furthermore, we analyzed the survival data and RNAseq data of 537 ccRCC patients from TCGA database (http://cancergenome.nih.gov) using LinkedOmics (http://linkedomics.zhang-lab.org/admin.php/) (Vasaikar *et al*., 2018). The results revealed that patients (50%) with higher ERβ mRNA expression had a significantly shorter overall survival (Fig. 1C). ERβ mRNA level was decreased along with the increased pathology T stage of tumors (Fig. 1D) and was significantly higher in the T3/T4 stage compared to the T1/T2 stage, and the metastasis patients had significantly higher ERβ expression compared to patients with non‐metastasis (Fig. 1E). The pathological and clinical information of those patients is shown in Table S1A.

Taken together, the results from multiple human RCC samples analyses (Fig. 1A–E) suggest that a higher ERβ expression is linked to the higher grades and stages of RCC and a worse survival.

### 3.2 Estrogen and ERβ play positive roles to promote RCC cell growth

**Figure 2:**
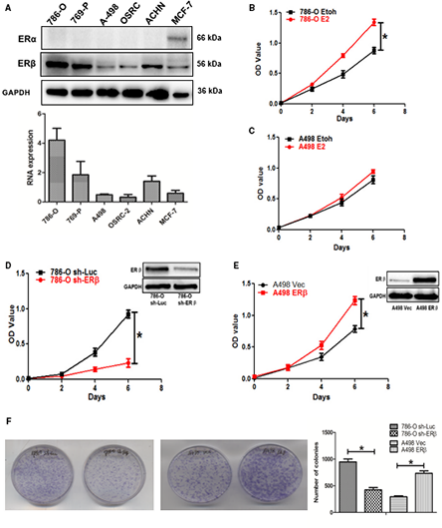
Comparison of ERβ expression in RCC cell lines and testing 17 β‐estradiol (E_2_) and ERβ effects on RCC cell growth. (A) Western blot (upper) and quantitative PCR (lower) detection of ERα and ERβ proteins and mRNAs in different RCC cell lines. Expression of ERα was not detected in tested RCC cells. ERβ has the highest expression level in 786‐O and the lowest in A498. Breast cancer MCF‐7 cells were used as a positive control. (B) Estrogen/ERβ effects on RCC cell growth. The growth rate of 786‐O cells (with high endogenous ERβ) is increased in the presence of 10 nm E_2_ compared to the control (EtOH). (C) Growth rate of A498 cells (with low endogenous ERβ) was not significantly changed by estrogen treatment. (D, E) Changes in ERβ expression levels can affect the RCC cell growth. We used lentivirus system to knock down ERβ in RCC 786‐O cells by lentiviral sh‐ERβ and used sh‐Luciferase (sh‐Luc) as a control, or to increase ERβ in A498 cells by lentiviral ERβ cDNA and used lentiviral vector as a control. Cell growth rates were determined by the MTT assay. (F) Colony formation assays showed ERβ knockdown in 786‐O could inhibit cell growth, and the overexpression of ERβ in A498 (with low endogenous ERβ) cells could increase cell growth (*P < 0.05).

We then validated the ERβ protein and mRNA expression in various RCC cell lines using western blot and quantitative PCR assays. The results showed higher ERβ expression in RCC 786‐O and 769‐P cells and lower ERβ expression in A498 and OSRC‐2 cells, using MCF‐7 cells as the positive control (Fig. 2A). By contrast, we found little expression of ERα in the five different RCC cell lines examined.

We then assayed the ERβ roles on RCC cell growth, and the results revealed that treatment with 10 nm 17β‐estradiol (E_2_) increased RCC cell growth in 786‐O cells (Fig. 2B) but not in A498 cells (Fig. 2C). In addition, knocking down ERβ with ERβ‐shRNA suppressed RCC cell growth in RCC 786‐O cells (Fig. 2D) and increasing ERβ with ectopic ERβ‐cDNA expression increased RCC cell growth in A498 cells (Fig. 2E). Similar results were also obtained when we replaced the MTT cell growth with the colony formation growth assay (Fig. 2F).

Taken together, the results shown in Fig. 2A–F obtained using multiple approaches in different RCC cell lines all demonstrate that E_2_/ERβ signals could play positive roles to promote RCC cell growth.

### 3.3 ERβ promotes RCC cell migration and invasion

**Figure 3:**
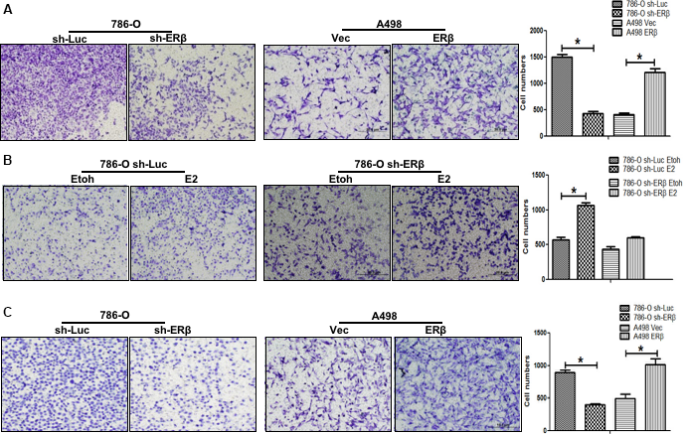
ERβ promotes the migration and invasion of RCC cells. (A) Higher ERβ expression promotes RCC cell migration. ERβ knockdown in 786‐O cells (786‐O sh‐ERβ) inhibits cell migration compared to 786‐O sh‐Luc cells. ERβ over‐expression in A‐498 cells (A‐498 ERβ) can increase cell migration capability. Quantification is shown on the right. (B) The migration rate of ERβ‐positive RCC cells (786‐O sh‐Luc) is increased further in the presence of E_2_. As a comparison, E_2_ treatment has little effect on the migration of 786‐O‐shERβ cells. (C) Higher ERβ expression promotes RCC cell invasion. ERβ knockdown in 786‐O cells (786‐O sh‐ERβ) inhibits cell invasion compared to 786‐O sh‐Luc cells. ERβ over‐expression in A‐498 cells (A‐498 ERβ) can increase the cell invasion capability. All experiments were performed in triplicate, and experiments were repeated at least three times independently. Quantifications of cell numbers that passed through the transwells were averaged from five representative fields (right). Data are the mean ± SD (*P < 0.05).

To examine the ERβ effect on RCC metastasis, we applied the transwell system to assay RCC cell migration and invasion. As shown in Fig. 3A, knocking down ERβ suppressed RCC 786‐O cell migration. Compared to mock treatment, treatment with 10 nm E_2_ could increase migration in the 786‐O sh‐Luc cells (Fig. 3B) and the effect is diminished in the ERβ knocked down 786‐O cells (786‐O‐shERβ) (Fig. 3B).

Similar results were also obtained when we replaced the cell migration with cell invasion using the matrigel transwell assay. Data showed that knocking‐down ERβ could suppress cell invasion in 786‐O cells (786‐O sh‐ERβ) (Fig. 3C). By contrast, adding functional ERβ led to an increased invasion in A498 cells (Fig. 3C). To prevent the non‐specific shRNA knockdown, we have constructed the second ERβ shRNA, sh‐ERβ#2. The results showed that sh‐ERβ#2 can also effectively knock down ERβ and reduce RCC invasion (Fig. S2).

Taken together, the results shown in Fig. 3A–C reveal that E_2_ could function through activation of ERβ to enhance RCC cell migration and invasion in different RCC cells.

### 3.4 ERβ alters cell morphology with increased epithelial–mesenchymal transition (EMT) and miRNA in RCC cells

**Figure 4:**
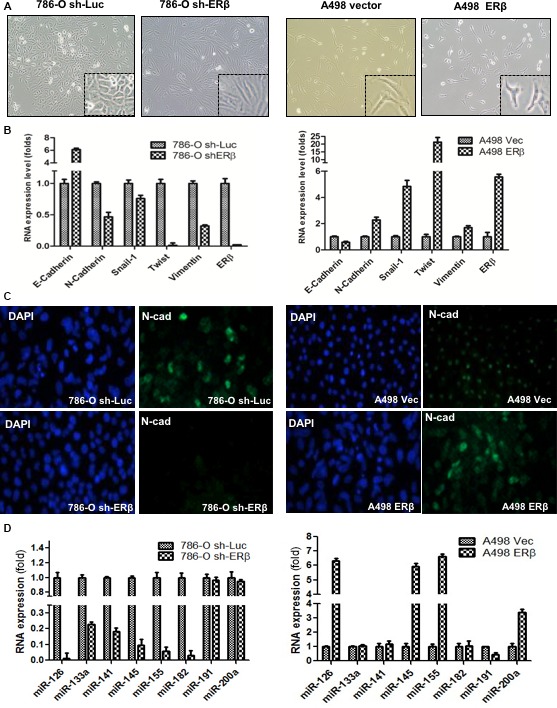
ERβ induces the EMT morphology and promotes EMT marker expression via miRNAs in RCC. (A) Changes of ERβ expression lead to correlative epithelial‐mesenchymal morphology changes in RCC cells. Cells became slender when ERβ is knocked down in 786‐O cells (sh‐ERβ vs. sh‐Luc), or became spindle ‐shaped with the addition of ERβ in A498 cells (A‐498 ERβ vs. A‐498 Vec). The correlative morphology changes suggest a mesenchymal‐like shape when RCC cells have higher ERβ levels. (B) Higher ERβ increases EMT marker expression in RCC. In 786‐O cells, we compared 786‐O sh‐ERβ vs. 786‐O sh‐Luc. In A498 cells, we compared A498 Vec vs. A498 ERβ. The higher ERβ expression and morphology changes are accompanied by the changed expression of EMT markers with increased levels of N‐cadherin, Snail, Twist and Vimentin, as well as reduced E‐cadherin. (C) Immunofluorescence staining of N‐cadherin in 786‐O and A498 cells with high vs. low ERβ. Nuclei were visualized by 4′,6‐diamidino‐2‐phenylindole staining. (D) Real‐time PCR of quantification of metastasis‐related miRNAs in RCC cells. Comparisons were made in 786‐O cells (sh‐ERβ vs. sh‐Luc) and A498 cells (ERβ vs. Vec). Data are the mean ± SD. Each experiment was independently repeated three times.

Interestingly, we noted the significant changes of cell morphology after altering ERβ expression, with cells becoming slender when ERβ was knocked down in 786‐O cells, or cells becoming spindle‐shaped with increasing ERβ expression in A498 cells (Fig. 4A). Importantly, we found that cell morphology changes were accompanied by the EMT marker changes, with decreased expression of N‐cadherin, Snail, Twist and Vimentin, as well as an increased E‐cadherin in 786‐O cells with shERβ (Fig. 4B, left). Consistantly, we observed increased expressions of N‐Cadherin, Snail, Twist and Vimentin accompanied by a decreased E‐cadherin in A498 cells with ERβ overexpression (Fig. 4B, right). As expected, increased ERβ led to increased N‐cadherin expression when comparing the 786‐O sh‐Luc vs. 786‐O sh‐ERβ and A498 ERβ vs. A498 Vec control (Fig. 4C). These results show that ERβ may function by altering the EMT to change RCC cell morphology. Because EMT could play key roles in the cell invasion (Bonnomet *et al*., 2012), these results further suggest that ERβ may function by altering the EMT to influence RCC cell invasion.

In addition to detecting the expression of EMT markers, we further examined a group of EMT/metastasis‐related miRNAs using a quantitative PCR assay and the results revealed that microRNAs (miRNA, miR) such as miR126, miR145 and miR155 were up‐regulated in RCC cells, with higher ERβ expression in both cell lines (Fig. 4D). Interestingly, as miR145 is the downstream effector of TGB‐β1 signals (Mayorga and Penn, 2012), these results suggest that E_2_/ERβ signals may function by altering the TGF‐β1 signals to increase RCC cell invasion.

### 3.5 TGF‐β1/SMAD3 as a key player for ERβ‐mediated RCC cell migration/invasion

To investigate the connection between ERβ‐increased RCC cell migration/invasion and the TGF‐β1 signals, we then applied quantitative PCR‐based focus‐array analyses to search for the key metastasis‐related genes that are responsible for ERβ‐enhanced RCC cell invasion. As expected, TGF‐β1 and SMAD3 are among those altered metastasis‐related genes (Table S1B) whose expression was increased in RCC cells when ERβ expression was elevated (A498: ERβ vs. vector, *P* < 0.01) and decreased in RCC cells when ERβ was knocked down (786‐O: shERβ vs. vector, *P* < 0.01).

**Figure 5:**
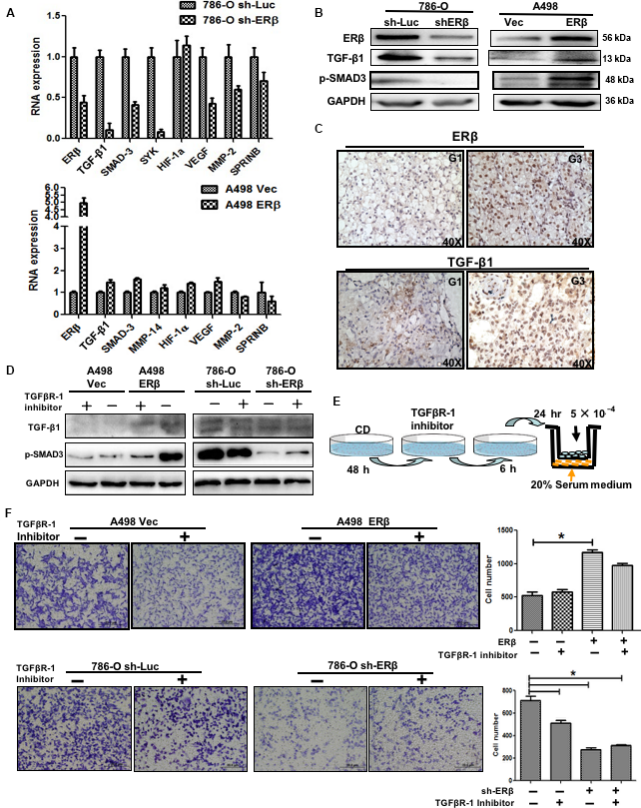
The TGF‐β1/SMAD3 signal was a key player for ERβ‐mediated proliferation and migration in RCC cells. (A) Quantitative PCR assay to screen metastasis‐related genes in 786‐O sh‐ERβ/sh‐Luc and A498 ERβ/Vec cells. Quantitative PCR results showed that higher ERβ expression could have higher mRNA levels of TGF‐β1 and SMAD3 in 786‐O sh‐Luc cells as compared to 786‐O sh‐ERβ cells (upper panel), and in A498 ERβ cells as compared to A498 Vec cells (lower panel). (B) Expression of ERβ, TGF‐β1 and SMAD3 protein levels in 786‐O sh‐ERβ/sh‐Luc (left) and A498 ERβ/vec cells (right) were detected by western blot analysis. (C) IHC staining of RCC clinical samples showed the correlative higher expression of ERβ and TGF‐β1 by comparing G3 vs. G1 (40× magnification, lower). (D) TGFβR‐1 inhibitor can reverse higher ERβ‐mediated signal changes. A western blot assay was applied to confirm the expression with TGF‐β1 and reduced pSMAD3 expression after treatment with TGFβR‐1 inhibitor in both cell lines. (E) Cartoon illustrating the experimental setting for treatment of RCC with TGFβR‐1 inhibitor and cell migration. RCC cells were cultured for 48 h and then treated with 10 μm TGFβR‐1 inhibitor for 6 h. RCC cells were then re‐seeded in the upper transwell (5 × 10^4^ per well) for 24 h to determine the migration rate. (F) TGFβR‐1 treatment can attenuate higher ERβ‐mediated RCC migration. The migration abilities of higher ERβ expressed RCC cells (786‐O sh‐Luc and A498 ERβ) were dramatically attenuated upon treatment with 10 μm TGFβR‐1 inhibitor for 48 h. Quantification of migrated cells was shown on the right panel. Data are the mean ± SD (*P < 0.05).

The results from the western blot and quantitative PCR assays also confirmed the above finding showing that altered ERβ expression could modulate the expression of TGF‐β1 and SMAD3 at mRNA (Fig. 5A) and protein levels (Fig. 5B) in both 786‐O and A498 cells. Furthermore, the results from human clinical sample surveys indicate that the higher ERβ expression is linked to the higher TGF‐β1 expression in higher Fuhrman's grade RCC tumors (Fig. 5C).

Importantly, the results from an interruption approach using TGFβR‐1 inhibitor for the cell invasion assay in both A498 and 786‐O cells (Fig. 5D) revealed that suppressing the TGF‐β1 signals with TGFβR‐1 inhibitor led to blockade of ERβ‐enhanced cell invasion in both 786‐O and A498 cells (Fig. 5E,F).

Taken together, the results shown in Fig. 5A–F obtained using multiple approaches indicate that ERβ can function by altering its downstream TGF‐β1 and SMAD3 to enhance RCC cell invasion.

### 3.6 ERβ promoted RCC cell growth and invasion in the *in vivo* mouse models

**Figure 6:**
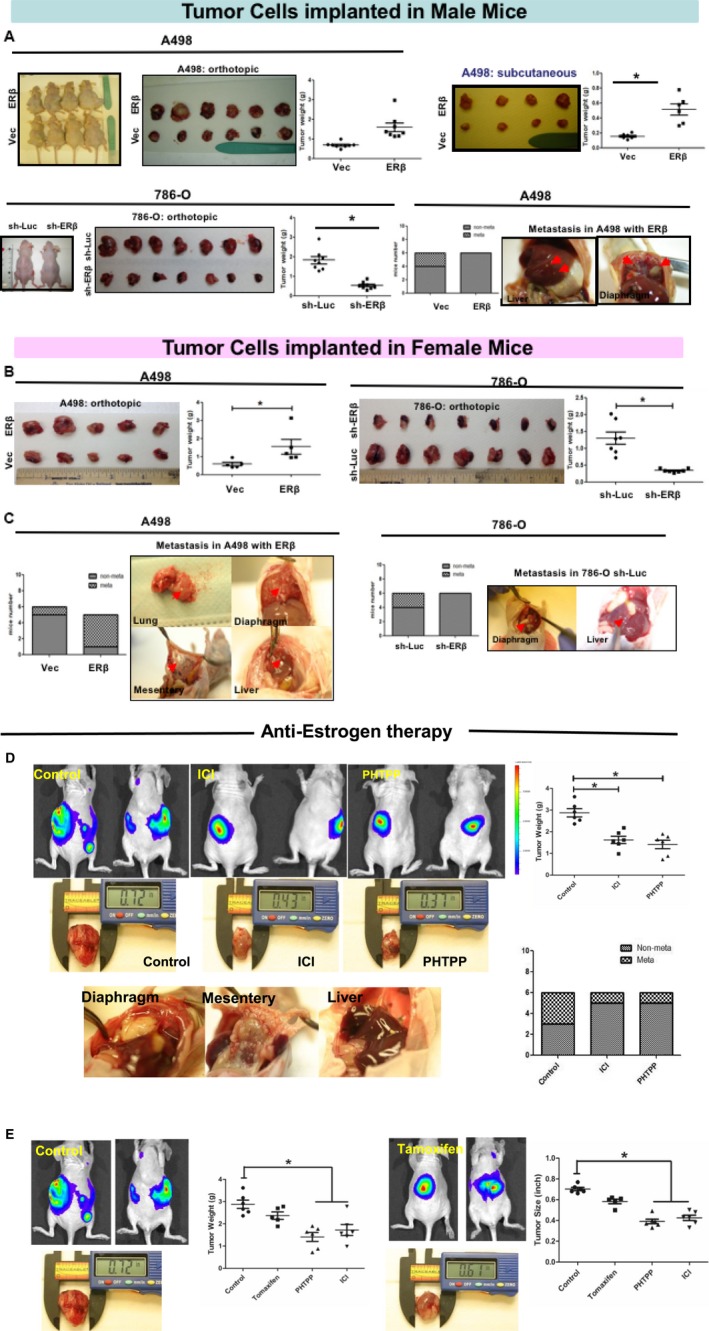
ERβ promotes RCC growth and metastasis in orthotopic or subcutaneous xenografted male and female mouse RCC models. (A) Orthotopic or subcutaneous implantation of RCC cells in male nude mice models. 2 pairs of RCC cells: A498 Vec/ERβ and 786‐O sh‐Luc/sh‐ERβ were orthotopically implanted under renal capsule or injected subcutaneously to 8‐week‐old male nude mice. One set of representative tumor growth data of subcutaneous implant A498 ERβ tumor data were presented (right, top panel). RCC tumor grafts with higher ERβ, A498‐O cells with ectopic ERβ cDNA expression, develop more distal metastatic tumors (right bottom panel). (*n *=* *6–7 mice per group, *P < 0.05). (B) Orthotopic or subcutaneous implantation of RCC cells in female nude mouse models. In 8‐week‐old female nude, RCC cells with high vs. low ERβ were implanted. The gross appearances, tumor volume and weight quantification were analyzed (*P < 0.05). The A498 ERβ cells develop a bigger tumor than the A498 vector controls. Similar results were also found in mice receiving 786‐O sh‐ERβ/sh‐Luc cells both in subcutaneous or orthotopic implantations. RCC tumor grafts with higher ERβ develop distal metastatic tumors (*P < 0.05). (C) Higher ERβ expression could stimulate RCC distal metastasis infemale mouse tumor models. Two out of six mice with A498 ERβ cells formed diaphragm, liver and hepatic hip lymph node metastasis and none of A498 Vec group had a metastasis. One out of six mice in 786‐O sh‐Luc formed distal metastasis, and none of 786‐O sh‐ERβ group had a metastasis. Representative metastasis images are shown, and red arrows indicate diaphragm and liver metastasis. (D) Anti‐estrogens, PHTPP and ICI 182,780 (ICI, Faslodex), inhibit RCC tumor growth and metastasis. IVIS imaging results show PHTPP and ICI inhibit the growth and metastasis of RCC *in vivo*. Mice tumor weights were measured (*P < 0.05). (E) Anti‐estrogen, tamoxifen (Nolvadex), was not as effective as ICI 182,780 (ICI; Faslodex) and PHTPP with respect to inhibiting RCC tumor growth. Compared to the mock control group, tamoxifen treatment could only reduce tumor weight, which is not as effective as PHTPP and ICI treatments on mouse RCC development *in vivo*. The results of IVIS imaging, primary tumor size, and tumor weights are shown and compared (*P < 0.05). Data are the mean ± SD.

To further confirm the above *in vitro* cell lines data with the *in vivo* mouse model, we implanted human RCC 786‐O cells with or without ERβ knockdown (786‐O sh‐ERβ/sh‐Luc) and A498 cells with or without ERβ over‐expression (A498‐ERβ/Vec) orthotopically and subcutaneously in male (Fig. 6A) and female mice (Fig. 6B and C).

RCC cells with higher ERβ (A498‐ERβ) could grow bigger tumors than cells with lower ERβ A‐498‐Vec in male mice (Fig. 6A, left top, orthotopically and right top, subcutaneously). Also, 786‐O sh‐Luc cells grew bigger tumors as compared to 786‐O sh‐ERβ (Fig. 6A, left bottom). Similarly, when implanted in female mice, those RCC cells with higher ERβ grew bigger tumors than those RCC cells with lower ERβ expression (Fig. 6B). As orthotopic impants and subcutaneous implants have similar results, we only showed a representative tumor growth data from subcutaneous implants in Fig. 6A.

We next examined the tumor metastasis rates. In the male group: two out of six mice with orthotopic implantation of A498 cells with over‐expressed ERβ (A498‐ERβ) had metastatic tumors in the diaphragm, liver and hepatic portal lymph nodes as compared to zero out of six mice with A498‐vec control implants (Fig. 6A, right bottom). In female animals, our results revealed that RCC cell xenografts with higher ERβ expression developed more distal metastatic tumors. Data showed that four out of five mice with orthotopically renal implanted A498‐ERβ cells formed diaphragm, liver, lung and mesentery metastasis and one of six mice had metastasis in A498‐vector control group (with lower ERβ) (Fig. 6C, left). Two out of six mice in the 786‐O sh‐Luc group formed distal metastasis, and none of the 786‐O sh‐ERβ group (ERβ knockdown) had metastasis (Fig. 6C, right). Histology analyses further confirmed those tumors are RCC cell tumors (Fig. S3A).

Together, our preclinical mouse RCC model data from both genders (Fig 6A–C), are consistent and supported by results from the TCGA database showing RCC patients with higher ERβ expression had shorter overall survival and disease‐free survival.

Furthermore, when comparing the same ERβ expression level of RCC implants in both male and female mice, we found that the tumor cells implanted in female mice developed tumors much earlier than tumor cells implanted in male mice. This could be a result of the higher estrogen concentrations in female mice. However, if we only compared ERβ expression within the male or female groups, RCC cell grafts with higher ERβ developed more distal metastatic tumors.

IHC staining of xenografted RCC tumors from nude mice demonstrated that RCC cells with a higher expression of ERβ have a higher expression of TGF‐β1 in the orthotopically xenografted RCC tumors when comparing A498 ERβ vs. A498 vector control cells and 786O sh‐ERβ vs. 786O sh‐Luc cells (Fig. S3B), suggesting that ERβ promotes RCC cell growth and invasion by altering the TGF‐β1 signal pathway.

Taken together, the results from *in vivo* mouse model show that higher ERβ expression can increase RCC metastasis by altering the TGF‐β1→SMAD3 signals using male mice or female mice tumor models.

### 3.7 Using anti‐estrogen PHTPP, ICI182,780 (Faslodex) and tamoxifen (Nolvadex) to suppress RCC cell growth and invasion in the *in vivo* mouse model

To develop the therapy and evaluate the above *in vitro* cell study and *in vivo* mouse data with respect to potential clinical use, we investigated the small molecules that can target this newly identified ERβ‐mediated signal pathway to better suppress RCC progression, and identified FDA‐approved anti‐estrogens ICI 182,780 (ICI, Faslodex) and tamoxifen (Nolvadex), as well as an ERβ selective antagonist PHTPP, which were all previously tested and shown to effectively inhibit estrogen induced ERβ transactivation with respect to ERE‐luciferase reporter activity.

Luciferase‐labeled 786‐O cells were implanted into the renal capsule of 8‐week‐old male mice (Fig. 6A) and female nude mice (Fig. 6B). At age 10 weeks, the mice were divided equally into different groups for treatment with DMSO, PHTPP and ICI. 10 μL of 1 × 10^−2^ m PHTPP, ICI or tamoxifen was mixed with 90 μL of sesame oil and injected intraperitoneally into each mouse, every other day. The IVIS Spectrum In Vivo Imaging System (PerkinElmer, Waltham, MA, USA) was used to monitor RCC tumor growth and distal metastasis. After 4 weeks of treatments, mice were sacrificed for tumor analysis.

The data showed that higher ERβ expression could stimulate RCC distal metastasis (Fig. 6C). Two out of six mice with A498 ERβ cells formed diaphragm, liver and hepatic hip lymph node metastasis and none of the A498 Vec group had metastasis. One out of six mice in 786‐O sh‐Luc formed distal metastasis and none of the 786‐O sh‐ERβ group had metastasis. Representative metastasis images reveal diaphragm and liver metastasis. The results show that anti‐estrogen treated mice had higher body weights than control mice. Overall, there are no obvious signs of toxicity or advert treatment side effects. All of the mice had developed RCC by the time of death. Importantly, we found that 50% mock treated mice developed metastasis, whereas only aproximately 17% of PHTPP or ICI treated mice developed distal metastasis (Fig. 6D). In addition to PHTPP and ICI, we also tested the effects of tamoxifen (Nolvadex) on mouse RCC growth. Because tamoxifen is not an effective antagonist for ERβ, the results indeed show that tamoxifen treatment is less effective at inhibiting RCC tumor (Fig. 6E).

Taken together, the preclinical study using *in vivo* mouse model confirms that targeting the newly identified ERβ/TGF‐β1/SMAD3 signals with the FDA‐approved anti‐estrogen ICI182,780 or the selective ERβ antagonist PHTPP could more effectively reduce RCC tumor growth and invasion.

## 4 Discussion

Estrogen is the main female hormone involved in various cell processes, including growth, differentiation and reproductive function. It interacts with two main types of ERs: ERα and ERβ. After binding to the receptors, estrogen exerts its genomic or non‐genomic functions via various signaling pathways. However, whether ERα or ERβ could affect RCC initiation or progression remains to be clarified. Using human renal tissue IHC staining, we found that ERα is negative, yet ERβ is positively located in the nuclei of RCC tissues, in both non‐cancerous renal cells and RCC cells. Interestingly, we found that ERβ expression increased, whereas RCC progressed to the later stages (T1 vs. T2‐3) or higher grades (G1 vs. G2‐3) (Fig. 1B). Although the ER protein could be detected in cytosol (Welsh *et al*., 2012), both ERα? and ERβ are predominantly nuclear and several studies have reported the nuclear staining of ERs (Dauvois *et al*., 1993; Roger et al., 2001; Miyamoto *et al*., 2012). Recently, a study investigated ERβ functions as a repressor for RCC progression (Yu *et al*., 2013), yet there were concerns regarding the cytosol staining of ERβ. We have tested several available antibodies for ERβ (including Abcam 14C8, GTX110607, SC 8974) and confirmed the specificity of antibodies using endogenous ERβ with or without shRNA knockdown. In addition, in cells without any endogenous ERs, we transfected ERβ into them to test its specificity. To carefully evaluate the clinical staining data, our laboratory has been working with two independent pathologists in the University of Rochester Medical Center and Johns Hopkins University to validate the specificity of ERβ antibodies. Through all of the validations, we have found that ERβ antibody (Abcam 14C8) could specifically recognize ERβ with nuclear staining signals. One early report stated that ERβ expression reduced higher grade RCC and plays an inhibiting role for RCC growth and invasion (Yu *et al*., 2013). However, the histology of the ERβ signal detected described in Yu *et al*. (2013) did not show clear nuclear staining. Also, the rabbit anti‐ERβ (Epitomics; Abcam) is not regarded as a specific anti‐ERβ antibody (Nelson *et al*., 2017). The mouse monoclonal antibody of ERβ that we used has been employed in several studies to detect ERβ in human tissues (Miyamoto *et al*., 2007, 2012; Rudolph *et al*., 2013). Another study by Chen *et al*. (2016) reported that estrogens inhibit RCC growth; however, the concentrations of E_2_ that they used to inhibit RCC growth were 12.5, 25 and 50 mm, which are 10 000–40 000‐fold higher than physiological concentrations. It is well known in the steroid field that super‐physiological concentrations of hormones can non‐specifically inhibit many types of cancer or non‐cancerous cells. Thus, the clinical significance of the data collected from RCC cells treated with super‐physiological concentrations of E_2_ remains unclear.

Our data of ERβ promoted RCC progression were validated using multiple strategies. In addition to IHC staining of clinical specimens, we have used multiple strategies and have purchased two different RCC cell lines from the ATCC. We have also used knockdown or ectopic overexpression of ERβ and *in vivo* and *in vitro* strategies, and all of the data obtained confirm that ERβ plays a promoting role in RCC growth and invasion.

It is well known that ERβ belongs to the nuclear receptor superfamily. Recently, several studies have shown that the membrane or cytoplasm ERβ existed and were functional (Levin, 2005). To determine the cellular location of ERβ, we have tested several available antibodies for ERβ and detected ERβ expression mainly in the nuclei. Importantly, we found that Erβ staining signals were mainly detected in the nuclei and its expression increased as RCC progresses to the later stages (T1 vs. T2‐3) or higher grades (G1 vs. G2‐3) (Fig. 1B). The results from TCGA database analysis showed that a higher ERβ expression level was associated with a worse survival rate. Although the most well‐known ERβ location is in the nuclei, membrane or cytoplasm ERβ have also been reported (Levin, 2005), although the staining signals are not yet sufficiently strong to be detected by standard methods and using a regular microscope. One previous study showed that the ERβ‐mediated PI3K/Akt pathway could regulate the cytoprotective effects of tocotrienol in a cellular Parkinson's disease model. Furthermore, ERβ has been demonstrated to interact with caveolin and form caveolae to activate cell survival signals (Nakaso *et al*., 2014). However, whether this ERβ signal pathway involves TGF‐β1 and SMAD3 pathways to regulate RCC progression remains to be investigated. Also, there is a truncated form of ERβ that can mediate the E_2_ induced non‐genomic pathway (Volpicelli *et al*., 2014). Taken together, although the major functions of ERβ remain in the nuclei in RCC, possible cytosol and membrane forms of ERβ may exist. The existance and potential functions of cytosol and membrane ERβ isoforms require further examination in the future.

Other than regulating TGF‐β1, some studies have shown that ERs could function as transmitters of signals from various growth factors including epidermal growth factor (EGF), insulin and insulin‐like growth factor (IGF)‐I (Newton *et al*., 1994) in ER‐positive cells transfected with an ERE‐containing reporter plasmid. Other than functioning as transmitters, ERα and ERβ could possibly regulate the expression or functions of those signal pathways. Interestingly, we observed that ERβ could selectively up‐regulate TGF‐β1 but not EGF, insulin or IGF‐1 expression in RCC cells (Fig. 4).

In the present study, we found that higher ERβ expression could up‐regulate TGF‐β1, which subsequently influences the function of the downstream gene, SMAD3. Thus, we further investigated the effect of ERβ on the TGF‐β1 signaling pathway in the promotion of tumor progression. The results showed that TGF‐β1 stimulation in 786‐O RCC cells (with high endogenous ERβ expression) resulted in the promotion of RCC progression. Our data shown in Fig. 5 demonstrate that there is a low level of TGF‐β1 in A‐498 (with low endogenous ERβ). Another consideration is that the transduction of lentiviral ERβ cDNA into A‐498 cells cannot extend beyond 60% of transduction efficiency; thus, the TGFβ is partially induced from the baseline. In 786‐O, there is high endogenous ERβ and a correlated high TGF‐β1 expression. When we knocked down ERβ using a shRNA strategy, the mRNA and protein expressions of ERβ is significantly reduced, although not completely. Because 786‐O cells have a high endogenous ERβ, shERβ knockdown could show a more prominent change (Fig. 5B).

The present study is the first report to show that ERβ can enhance TGF‐β1/miR145, SMAD3 and the EMT pathway to control RCC invasion both *in vitro* and *in vivo*. Based on the IHC results obtained in RCC samples, we hypothesize that high ERβ expression plays a promoting role in RCC. The low level of ERβ expression generally found in low grade tumors may predispose them to additional carcinogen exposure and, in this way, contribute to possible tumor progression. Importantly, in the later stage, ERβ levels were observed to be elevated in a significant proportion of high‐grade tumors compared to low‐grade tumors. Thus, ERβ could also represent a marker of RCC progression. Our study has linked the ERβ signal with TGFβ1/miR145 and EMT signal pathways, which have high clinical significance.

In an earlier report, a multiple centers clinical trial used the ER antagonist tamoxifen to treat metastatic RCC. Some research centers have reported that the tamoxifen treatment can affect the prognosis of RCC patients (Al‐Sarraf *et al*., 1980), whereas others showed that treatment does not influence RCC patient progression (Papac and Keohane, 1993). In clinical trials in the 1990s, the results showed there was no significant therapeutic efficacy of the anti‐estrogen tamoxifen in advanced RCC (Henriksson *et al*., 1998). The present study indeed provides a possible explaination as to why tamoxifen treatment may not have satisfactory efficacy for RCC patients. Our data clearly show that some RCC tissues are ERβ‐negative (or low expression) and some are ERβ‐positive with high expressionusing IHC staining and a PCR assay (Figs 1 and S1A). Consistently, ERα is negative in RCC cell lines and human tissues (Fig. S1B). Therefore, anti‐estrogen therapy should be only applied to ERβ(+) RCC patients. Furthermore, tamoxifen is not the most effective antagonist for ERβ. Comparing the results shown in Fig. 6D for ICI 182,780 (40.3% reduction) and PHTPP (51% reduction), tamoxifen only has 17.8% efficacy with respect to the reduction of ERβ(+) RCC tumor weight (Fig. 6E). Taken together, our study could provide new insight to facilitate the redesign of the use of anti‐estrogen therapy on ERβ(+) RCC patients. A summary diagram of the ERβ regulated TGF‐β1/SMAD3/miRNAs/EMT pathways to control growth and invasiveness of RCC is provided in Fig. S4.

## Conclusions

In the present study, we provide evidence indicating that ERα is undetectable in the kidney or RCC tissues, and that there is an increased ERβ expression in the tumors at later stages or higher grades of RCC. TCGA database analysis showed that the increased ERβ is associated with a worse survival for RCC patients. Further analyses using *in vitro* cell studies and pre‐clinical mouse RCC models showed that estrogens function via ERβ to promote the proliferation, migration and invasion of RCC. In addition, our data confirmed that ERβ affected the expression of TGF‐β1/SMAD3 signals to control RCC invasion. Targeting ERβ/TGFβ1/SMAD3 signals with selective estrogen receptor modulators (e.g. a FDA‐approved anti‐estrogen, Faslodex, or an ERβ selective antagonist, PHTPP) could help in the development of new therapies to better treat RCC patients.

## Author contributions

WS designed and performed the experiments and wrote the manuscript. YC provided suggestions and participated in the data analysis. C‐RY and IH contributed to the development of methodology and the mouse model experiment. QH and XZ contributed to human renal specimen collection and pathological diagnoses. LZ and JC contributed to clinical data analysis. LS‐SC, KMD and CC provided intellectual input and helped with the writing of the manuscript. DH, LL and SY conceived the study, designed the experiments, and edited and approved the final version of the manuscript for publication.

## Supporting information


**Fig. S1.** Detection of ERβ mRNA expression in different RCC grades (G) of RCC and confirmation that ERα expression was negative in human RCC tissues.
**Fig. S2.** Validation of estrogen/ER signals on RCC invasion using specificity of shERβ knockdown by the second sh‐Erβ, as well as estrogen treatment, to test the roles of E_2_/ERβ with respect to RCC migration in A498 cells with or without ectopic ERβ cDNA expression.
**Fig. S3.** (A) Histological image analysis for primary and metastatic RCC tumors. (B) IHC results of TGFβ‐1 signals in 786‐O sh‐Luc vs. 786‐O sh‐Erβ, as well as in A498 Vec vs. A498 ERβ.
**Fig. S4.** ERβ regulates TGFβ‐1/SMAD/MiRNAs/EMT pathways to control growth and invasiveness of RCC cells.
**Table S1.** Investigation of the expression of ERs in human RCC tissue, as well as the ER signal regulated invasion and metastasis gene transcription profile in RCC cells, using a focused quantitative PCR array.
