# Supplementary material for: Targeting newly identified ERβ/TGF‐β1/SMAD3 signals with the FDA‐approved anti‐estrogen Faslodex or an ERβ selective antagonist in renal cell carcinoma
Source: Mol Oncol. 2018 Oct 30;12(12):2055–71. doi: 10.1002/1878-0261.12377 (PMC6275262; doi:10.1002/1878-0261.12377)
Supplement: Supplementary file 1 — Fig. S1. Detection of ERβ mRNA expression in different RCC grades (G) of RCC and confirmation that ERα expression was negative in human RCC tissues. Fig. S2. Validation of estrogen/ER signals on RCC invasion using specificity of shERβ knockdown by the second sh‐Erβ, as well as estrogen treatment, to test the roles of E2/ERβ with respect to RCC migration in A498 cells with or without ectopic ERβ cDNA expression. Fig. S3. (A) Histological image analysis for primary and metastatic RCC tumors. (B) IHC results of TGFβ‐1 signals in 786‐O sh‐Luc vs. 786‐O sh‐Erβ, as well as in A498 Vec vs. A498 ERβ. Fig. S4. ERβ regulates TGFβ‐1/SMAD/MiRNAs/EMT pathways to control growth and invasiveness of RCC cells. Table S1. Investigation of the expression of ERs in human RCC tissue, as well as the ER signal regulated invasion and metastasis gene transcription profile in RCC cells, using a focused quantitative PCR array. [file MOL2-12-2055-s001.pdf]

A. Summary of pathological and clinical data

Characteristics	Informative cases
Median age (range), years	63.2 (33–82)
Sex	
Male	52
Female	28
Stage	
T1	49
T2–3	31
Grade	
G1	33
G2-G3	47
Metastasis	
M0	74
M1	6
Surgical procedure	
Partial nephrectomy	36
Radical nephrectomy	44

M0, tumor without metastasis;
M1, tumor with metastasis;

B. Q-PCR array data on 780-O sh-ERβ/sh-Luc and A498 ERβ/vec

786-O	Sh-ERβ /sh-Luc (fold)
MMP14	1.47191
MMP2	1.66362
TGFβ-1	9.15372
SMAD3	2.65357
SPRINB	1.42320
EZH2	0.78935
MTA1	1.35484
DAB2IP	0.97325
KAI1	1.75562
HIF2a	0.87354
VEGFa	2.32787

A498	ERβ/Vec (fold)
MMP14	1.20765
MMP2	1.58296
TGFβ-1	1.26886
SMAD3	1.65716
SPRINB	0.61334
EZH2	0.5933
MTA1	0.30670
DAP2IP	1.34886
KAI1	1.27308
HIF2a	0.80389
VEGFa	1.48193

A

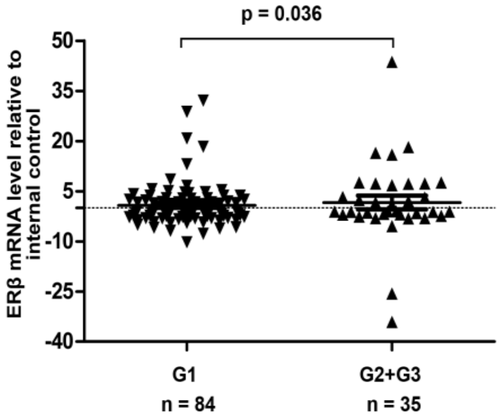
p = 0.036
ERβ mRNA level relative to internal control
50
35
20
5
-10
-25
-40
G1
n = 84
G2+G3
n = 35

B

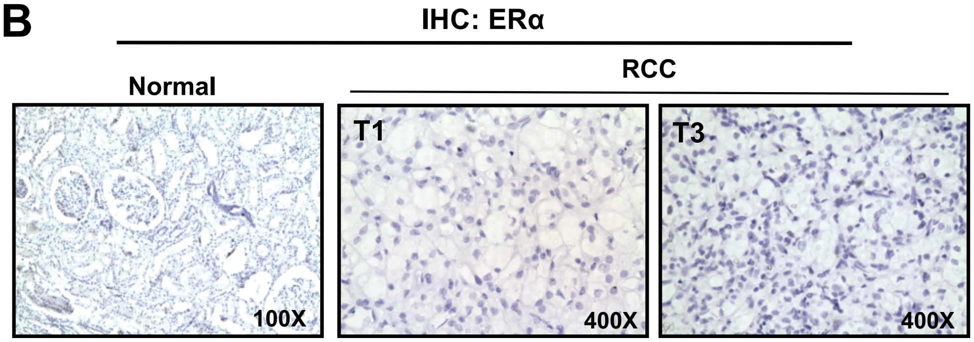
IHC: ERα
Normal
RCC
T1
T3
100X
400X
400X

Song *et al.*, Supplemental Fig. 2

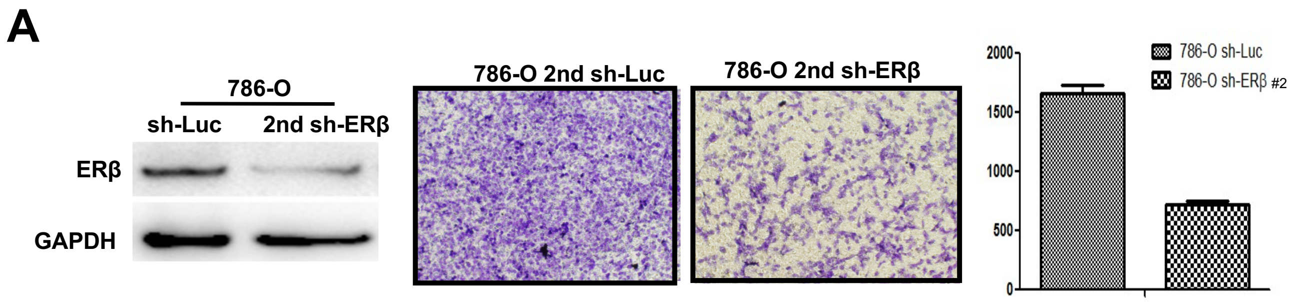

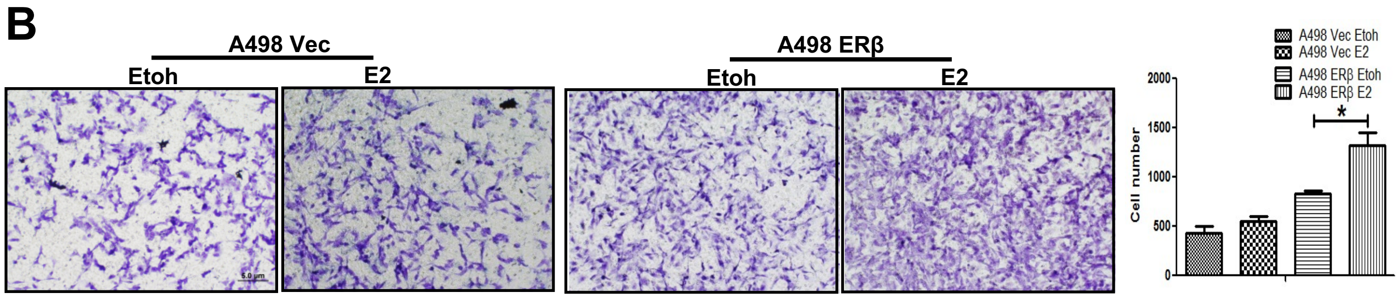

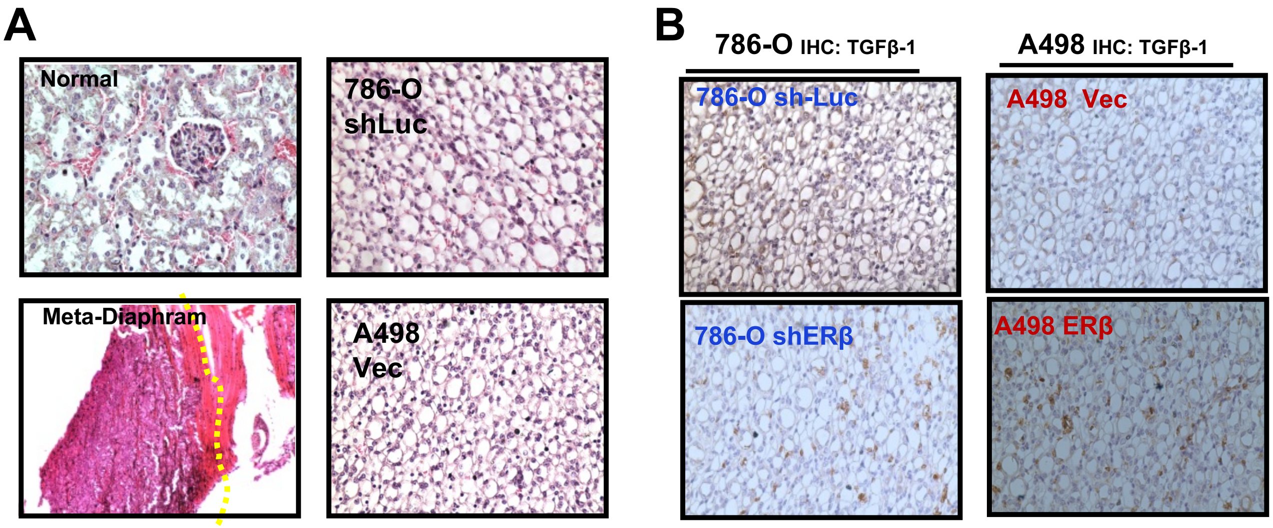
A
Normal
786-O
shLuc
Meta-Diaphram
A498
Vec
B
786-O IHC: TGFβ-1
A498 IHC: TGFβ-1
786-O sh-Luc
A498 Vec
786-O shERβ
A498 ERβ

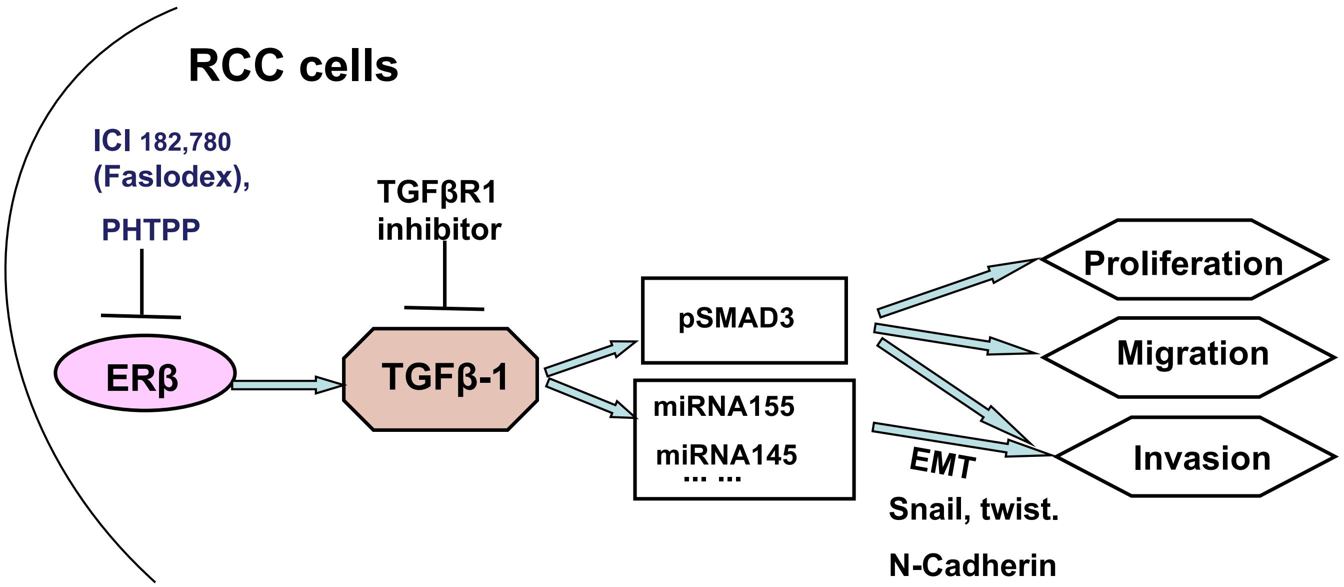
RCC cells
ICI 182,780
(Faslodex),
PHTPP
TGFβR1
inhibitor
ERβ
TGFβ-1
pSMAD3
miRNA155
miRNA145
... ...
Proliferation
Migration
Invasion
EMT
Snail, twist.
N-Cadherin
